# Mad3 KEN Boxes Mediate both Cdc20 and Mad3 Turnover, and Are Critical for the Spindle Checkpoint

**DOI:** 10.1371/journal.pone.0000342

**Published:** 2007-04-04

**Authors:** Emma M.J. King, Sjaak J.A. van der Sar, Kevin G. Hardwick

**Affiliations:** Wellcome Trust Centre for Cell Biology, University of Edinburgh, Edinburgh, United Kingdom; Fred Hutchinson Cancer Research Center, United States of America

## Abstract

Mitotic progression is controlled by proteolytic destruction of securin and cyclin. The mitotic E3 ubiquitin ligase, known as the anaphase promoting complex or cyclosome (APC/C), in partnership with its activators Cdc20p and Cdh1p, targets these proteins for degradation. In the presence of defective kinetochore-microtubule interactions, APC/C^Cdc20^ is inhibited by the spindle checkpoint, thereby delaying anaphase onset and providing more time for spindle assembly. Cdc20p interacts directly with Mad2p, and its levels are subject to careful regulation, but the precise mode(s) of APC/C^ Cdc20^ inhibition remain unclear. The mitotic checkpoint complex (MCC, consisting of Mad3p, Mad2p, Bub3p and Cdc20p in budding yeast) is a potent APC/C inhibitor. Here we focus on Mad3p and how it acts, in concert with Mad2p, to efficiently inhibit Cdc20p. We identify and analyse the function of two motifs in Mad3p, KEN30 and KEN296, which are conserved from yeast Mad3p to human BubR1. These KEN amino acid sequences resemble ‘degron’ signals that confer interaction with APC/C activators and target proteins for degradation. We show that both Mad3p KEN boxes are necessary for spindle checkpoint function. Mutation of KEN30 abolished MCC formation and stabilised Cdc20p in mitosis. In addition, mutation of Mad3-KEN30, APC/C subunits, or Cdh1p, stabilised Mad3p in G1, indicating that the N-terminal KEN box could be a Mad3p degron. To determine the significance of Mad3p turnover, we analysed the consequences of *MAD3* overexpression and found that four-fold overproduction of Mad3p led to chromosome bi-orientation defects and significant chromosome loss during recovery from anti-microtubule drug induced checkpoint arrest. In conclusion, Mad3p KEN30 mediates interactions that regulate the proteolytic turnover of Cdc20p and Mad3p, and the levels of both of these proteins are critical for spindle checkpoint signaling and high fidelity chromosome segregation.

## Introduction

During mitosis and meiosis cells segregate their replicated genomes to daughter nuclei [1]. Any errors will result in aneuploidy, which typically leads to disease or cell death [2]. Cells employ surveillance systems, referred to as checkpoints, to ensure that their genomes are replicated, repaired and segregated with high fidelity. The spindle checkpoint delays anaphase onset until all sister chromatid pairs are bi-oriented on the mitotic spindle [3]–[5]. Mitotic progression and exit are regulated to a large extent by ubiquitin-dependent proteolysis. The major mitotic E3 ubiquitin ligase is known as the anaphase promoting complex or cyclosome (APC/C, [6]). The APC/C requires the action of an activator: early in mitosis this is Cdc20p and later in mitosis and in G1 it switches to Cdh1p [7]. Cdc20p is the key target of the spindle checkpoint [8], [9], and its abundance and activity are regulated at many levels including transcription, post-translational modification and proteolysis [10]–[12]. Cdc20p is both an APC/C activator and an APC/C substrate [13], and it was recently demonstrated that levels of Cdc20p are very precisely regulated in budding yeast mitosis [14]. If there is too much Cdc20p in the cell, then the spindle checkpoint is unable to inhibit it [8], [14].

APC/C substrates including securin (Pds1p) and cyclin (Clb2p) contain recognition signals, typically known as destruction or D boxes, and/or KEN boxes [15], [16]. Once poly-ubiquitinated by the APC/C, the D or KEN box containing protein is degraded by the proteasome. It is generally thought that KEN boxes are recognised by the Cdh1p activator, rather than Cdc20p which preferentially recognises D boxes [17]–[19]. However, this is probably an over-simplification and it has also been shown that the vertebrate APC/C is capable of recognising destruction motifs directly, in both a Cdc20p and Cdh1p-independent manner [20].

Models of the spindle checkpoint mechanism have been significantly enhanced by information derived from structural Mad2 studies [21], [22], and dynamic imaging (FRAP) of the checkpoint proteins and Cdc20p [23]–[26]. These studies have lead to models in which a dynamic pool of Mad2p interacts with a stable Mad2p-Mad1p “template” at the kinetochore. After kinetochore transit, the dynamic Mad2p is released in a form that efficiently interacts with and inhibits Cdc20p [22], [27]. One thing lacking from these models is a clear role for the Mad3p/BubR1 checkpoint component. Not only are these proteins required for normal spindle checkpoint function [28]–[30], but in fission yeast we have shown that Mad3p is even required for the overexpression of Mad2p to induce a metaphase arrest [31]. Such results indicate that Mad2p-Cdc20p complex formation is unlikely to be sufficient for checkpoint arrest *in vivo*. It has also been shown that the MCC (Bub3-BubR1-Mad2-Cdc20) is a far more potent inhibitor than Mad2p *in vitro* [29], [32], [33].

Here we investigate the role(s) of Mad3p in Cdc20p inhibition, and in particular focus on the two conserved KEN boxes in Mad3p, both of which we show to be necessary for spindle checkpoint function. Importantly, mutation of the N-terminal Mad3p KEN box (*mad3-KEN30AAA*) also completely prevents Cdc20p binding. This mutation blocks MCC formation, dramatically reduces Cdc20p turnover in mitosis, and has a checkpoint null phenotype. We propose that this N-terminal KEN box, which is conserved from yeast Mad3p to human BubR1, is a critical link required for MCC formation and spindle checkpoint inhibition of Cdc20p.

In addition, we propose that the N-terminal KEN box acts as a degron during G1, leading to Mad3p turnover in a Cdh1-APC/C dependent manner. We analyse the effects of Mad3p overproduction and observe chromosome bi-orientation defects and significant chromosome loss during the recovery from checkpoint arrest.

## Results

### Mad3p contains two conserved KEN boxes

**Figure 1:**
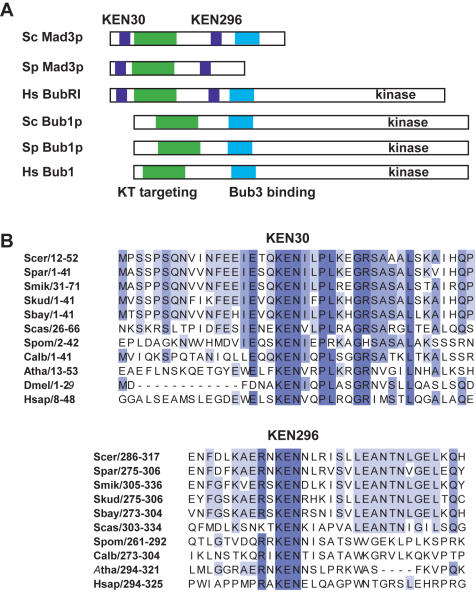
Mad3p contains two conserved KEN boxes. A) Mad3/BubR1 domain structure–schematic diagrams of the Mad3 and Bub proteins indicate the organisation of their functional domains. B) Clustal×alignment of the two conserved KEN boxes found in Mad3/BubR1. Species indicated are *Saccharomyces cerevisiae* (Scer), *Saccharomyces paradoxus* (Spar), *Saccharomyces mikatae* (Smik), *Saccharomyces kudriavzevii* (Skud), *Saccharomyces bayanus* (Sbay), *Saccharomyces castellii* (Scas), *Schizosaccharomyces pombe* (Spom), *Candida albicans* (Calb), *Arabidopsis thaliana* (Atha), *Drosophila melanogaster* (Dmel) *and Homo sapiens* (Hsap). Numbers indicate residue position within protein sequence.

Mad3p, and its vertebrate homologue BubR1, contain a highly conserved KEN box motif near their N-termini. Indeed this is used to distinguish BubR1 from the related, but functionally distinct, checkpoint component Bub1p [34]. The Mad3/BubR1 family also contain a second KEN box (Fig 1A). The function(s) of these motifs in Mad3/BubR1 has not previously been addressed.

**Figure 2:**
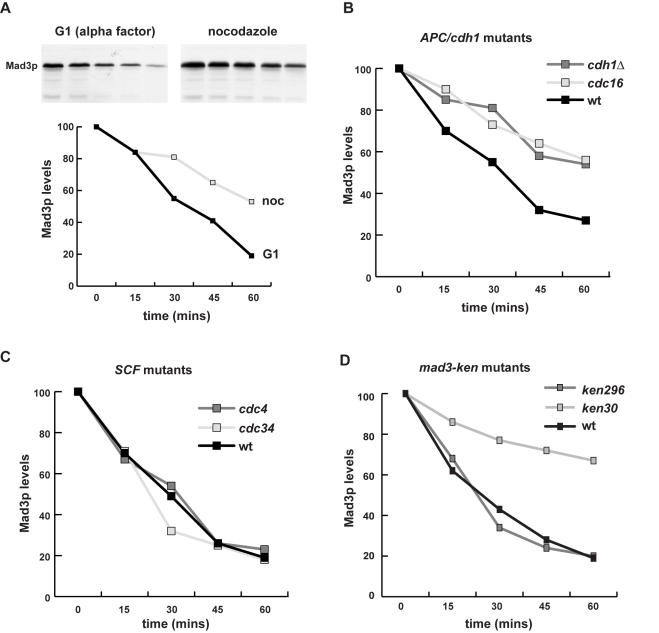
Mad3p is an APC/C substrate. A) Mad3p is unstable in G1. Cells expressing *GAL-MAD3* were first synchronised in G1 (with alpha factor) or mitosis (with nocodazole) in raffinose media, and then a pulse of Mad3p expression was induced by 30 minutes of growth in media containing 2% galactose. Cells were then washed with glucose media (YPD) and cycloheximide was added to inhibit new protein synthesis. The G1 and mitotic arrests were maintained, samples taken at the times indicated, and whole cell extracts immunoblotted with anti-Mad3p antibodies to determine the remaining level of Mad3p. B) Mad3p turnover in G1 is APC/C dependent. Cells expressing *GAL-MAD3* were synchronised in G1 (with α-factor) in raffinose media, and then a pulse of Mad3p expression was induced by 30 minutes of growth in media containing 2% galactose. Cells were then shifted to 36°C (restrictive temperature for *cdc16*-ts) for 30 minutes, washed with glucose media (YPD) and cycloheximide was added to inhibit new protein synthesis. The G1 arrest was maintained, samples taken at the times indicated, and whole cell extracts immunoblotted with α-Mad3p antibodies to determine the remaining level of Mad3p. C) SCF mutants do not stabilise Mad3p in G1. *cdc4* and *cdc34* strains expressing *GAL-MAD3* were analysed for Mad3p turnover as in B. D) *mad3KEN30AAA* stabilises Mad3p in G1. *mad3Δ* cells containing GAL driven wild-type or *mad3-ken* mutants were arrested in alpha factor, and then Mad3p was induced for 30 minutes with galactose media. Cells were then washed in glucose media and cycloheximide was added to prevent new protein synthesis. Samples were taken at the times indicated, and whole cell extracts immunoblotted with anti-Mad3p antibodies for the level of Mad3p.

Our previous studies suggested that the steady state level of Mad3p was relatively constant through the budding yeast cell cycle [28]. To investigate whether Mad3p was degraded by APC/C-dependent proteolytic turnover we analysed its stability, in mitosis and in G1. To do this we synchronised cells, added cycloheximide to prevent new protein synthesis, and then analysed the level of Mad3p through a time course using polyclonal anti-Mad3p antibodies. The levels of Mad3p after a prolonged G1 arrest were rather low (not shown), presumably because much of it had been degraded. Therefore, we repeated this experiment after a pulse of *MAD3* expression from the strong inducible *GAL1* promoter. Mad3p levels fell slowly in nocodazole, and significantly faster in G1 during an alpha factor arrest (Fig. 2A). As the APC/C is very active in G1 this finding is consistent with APC-dependent turnover and Mad3p being a late mitotic/G1 substrate. If the Mad3p KEN boxes act as destruction signals recognised by the APC/C, Mad3p should be stabilised in conditional (ts) alleles of APC/C components, and possibly in the absence of the late APC/C activator, Cdh1p. To test this we carried out cycloheximide chase experiments in G1 arrested cells in *cdc16-123* (apc-ts) and *cdh1Δ*mutants, along with *cdc4-1* and *cdc34-2* strains as controls. The latter are ts alleles of components of another E3 ubiquitin ligase, the SCF [35]. All strains expressed Mad3p from the strong inducible *GAL1* promoter. The *cdh1* and *cdc16* mutations both stabilised Mad3p (Mad3p stabilisation was also observed in *cdc23-1*–data not shown), whereas *cdc34* and *cdc4* did not, demonstrating that Mad3p is degraded in a Cdh1-APC/C-dependent manner (Fig. 2B/C).

To analyse the role of the KEN boxes in Mad3p turnover we replaced them, either individually or in combination, with triple alanine (AAA) residues. When we analysed the stability of these mutant Mad3 proteins in G1 arrested cells there was little difference in mad3-KEN296AAAp stability when compared to wild-type Mad3p, but Mad3-KEN30AAAp was clearly stabilised (half-life of ∼90 minutes cf. 25 minutes for wild-type, Fig. 2D). Thus Mad3p turnover in G1 is dependent on Cdh1-APC/C activity and its own N-terminal KEN box (KEN30).

### Mad3p KEN boxes have checkpoint functions

**Figure 3:**
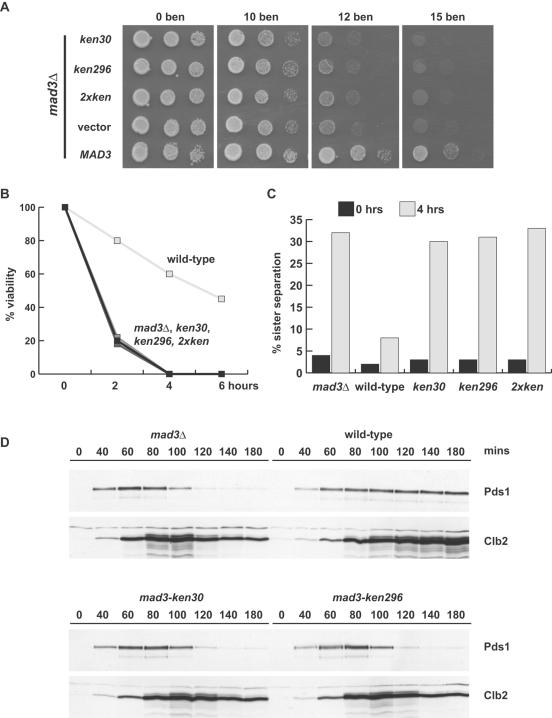
*mad3-KEN-AAA* mutants are spindle checkpoint defective. A) The *mad3-ken* mutants failed to complement the benomyl sensitivity of *mad3Δ*. The constructs indicated were transformed into the *mad3Δ* strain, and then diluted and plated onto YPD plates containing the indicated concentration of benomyl (µg/ml). Photos were taken after three days growth at 24°C. B) The *mad3-ken* mutants died rapidly. The strains from above were grown to log phase, synchronised in G1 with α-factor, released and then nocodazole was added to 20 µg/ml. At the times indicated, cells were taken, diluted, plated out and scored for their ability to form colonies on rich media. C) The *mad3-ken* mutants failed to maintain sister chromatid cohesion. In parallel these strains, which contain GFP-marked chromosomes, were scored for sister chromatid cohesion at 0 (α-factor arrested) and four hours after release into nocodazole-containing media. D) The *mad3-ken* mutants are unable to prevent securin and cyclin from being degraded. Similar strains to those above, but containing Pds1-Myc_13 _were synchronised in α-factor, then released into nocodazole media (20 µg/ml). Time points were taken, whole cell extracts made and immunoblotted for Pds1 (anti-myc) and cyclin levels (anti-Clb2p).

Next we analysed the effect of KEN box mutations on spindle checkpoint function(s) of Mad3p. When tested for their ability to complement a *mad3Δ* strain, all three mutants (KEN30AAA, KEN296AAA and the double mutant) were sensitive to the anti-microtubule drug benomyl. Their sensitivity was similar to that of the *mad3* null mutant (*mad3Δ*, Fig. 3A). Checkpoint assays were also carried out in liquid media containing nocodazole to depolymerise microtubules. These revealed that the *ken* mutants were unable to maintain sister-chromatid cohesion, scored by imaging GFP-marked chromosome IV, and died just as rapidly as the *mad3Δ* in the presence of nocodazole (Fig. 3B/C). Time courses and immunoblotting demonstrated that the *mad3-ken* mutants were unable to stabilise key mitotic regulators, Pds1p (Securin) and Clb2p (Cyclin B), when cells were challenged with nocodazole (Fig. 3D). Thus both Mad3 KEN boxes are critical for spindle checkpoint function.

### The N-terminal Mad3 KEN box is required for Cdc20p binding and for mitotic Cdc20p turnover

**Figure 4:**
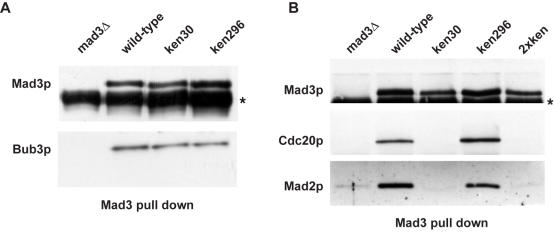
*mad3-KEN30AAA* fails to form a mitotic checkpoint complex (MCC). A) *mad3-KEN-AAA* mutants can bind Bub3p. Native extracts were made from the indicated strains, containing Bub3-myc_13_, and Mad3p complexes were immunoprecipitated then immunoblotted for Mad3p and Bub3p (anti-myc). B) *mad3-KEN30AAA* mutants don't bind to Mad2p or Cdc20p. Cells were arrested in mitosis (with hydroxyurea and nocodazole) and native extracts were made from the indicated strains, containing Cdc20p-myc_13_. Mad3p was immunoprecipitated then immunoblotted for Mad3p, Mad2p and Cdc20p (anti-myc).

Two clear modes of action of Mad3p have been described in budding yeast, both relating to inhibition of Cdc20p: mitotic checkpoint complex (MCC, which in budding yeast is Bub3p-Mad3p-Mad2p-Cdc20p) formation [28] and Cdc20p turnover [14]. Co-immunoprecipitation experiments demonstrated that neither KEN box mutation affected Bub3p complex formation (Fig. 4A), from which we conclude that the mutations have had little effect on the overall folding or stability of Mad3p. Importantly, the *KEN30AAA* mutation completely abolished the binding of Mad3p to both Cdc20p and Mad2p. This was not the case for *KEN296AAA* (Fig. 4B), although in some experiments a minor reduction in MCC levels was observed. Thus KEN30 is required for MCC assembly and/or stability.

**Figure 5:**
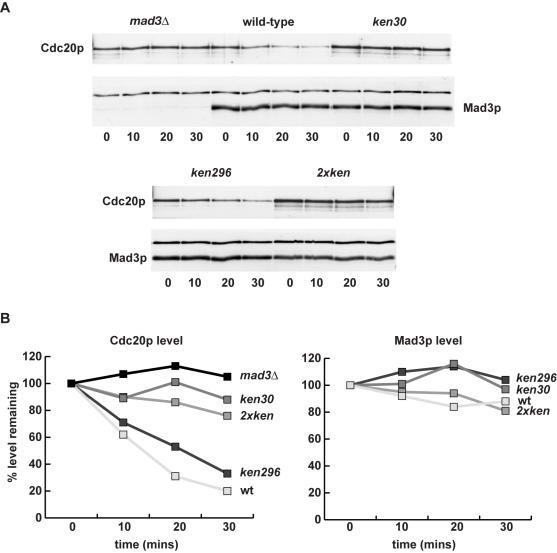
*mad3-KEN30AAA* stabilises Cdc20p in mitosis. A) Strains were arrested in mitosis (with nocodazole and hydroxyurea), and cycloheximide was added (time 0) to prevent new protein synthesis. Time points were taken and immunoblotted for Cdc20p levels (anti-myc) and Mad3p levels. B) Quantitation of the Cdc20p and Mad3p levels.

Surprisingly, Cdc20p turnover in early mitosis was shown to be independent of its own destruction boxes, but dependent on the Mad proteins [14]. We have found that all of the *mad* and *bub* mutations perturb Cdc20p turnover, and lead to a range of increased stability with *mad3Δ* having the most profound effect (data not shown). We were particularly interested to test whether the Mad3p KEN boxes had a role to play in Cdc20p turnover. To do this we synchronised cells early in mitosis (in several ways–see Materials and Methods), added cycloheximide to prevent new protein synthesis, and then took time points to analyse the Mad3p and Cdc20p levels in whole cell extracts. Whilst any effect on Mad3p turnover was subtle in these mitotic time courses, the effects on Cdc20p turnover were very pronounced (Fig. 5A/B). *KEN30AAA* led to stabilisation of Cdc20p, similar to that seen in a *mad3Δ* background (∼6-fold stabilisation), whereas *KEN296AAA* had little effect (Fig. 5C). Thus in two biochemical assays *mad3-KEN30AAA* behaved like the *mad3Δ* strain: there was no MCC complex and Cdc20p was stabilised. Either or both of these effects could explain its checkpoint-defective phenotype. *Mad3-KEN296AAA* did not significantly reduce Cdc20p binding nor stabilise Cdc20p, whereas the double KEN mutant behaved like *KEN30AAA* in all assays tested. These results demonstrate that Mad3p binding is necessary for Cdc20 turnover, and suggest the intriguing possibility that the N-terminal Mad3 KEN box acts *in trans* as a Cdc20p destruction signal during early mitosis.

### Mad3 overexpression causes bi-orientation defects

We have demonstrated that Mad3p is degraded late in mitosis and G1 in a Cdh1-APC/C dependent manner. To understand why Mad3p is subject to such regulation we decided to analyse the effects of Mad3p overexpression.

**Figure 6:**
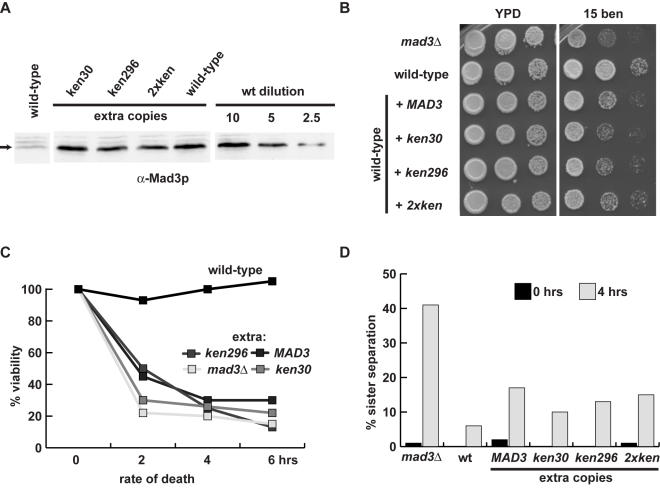
Over-expression of Mad3p induces micro-tubule drug sensitivity, but only subtly perturbs the spindle checkpoint. A) Strains expressing extra integrated copies of wild-type *MAD3* or ken mutant *mad3* were immunoblotted to quantitate their expression levels. B) Overexpression of Mad3p induces benomyl sensitivity. The over-expression strains were diluted and plated onto rich YPD media with or without the addition of 15 µg/ml of benomyl. Plates were photographed after 3 days growth at 24°C. C) Strains overexpressing Mad3p die quickly in the presence of nocodazole. The indicated strains were pre-synchronised in G1 with alpha factor, released into media containing 20 µg/ml nocodazole, and cells were plated out at indicated times. Viability was scored as the percentage of colonies formed relative to the zero time point. D) Mad3p overexpression has a mild effect on sister-chromatid cohesion. The overexpression strains, which containing GFP marked chromosome V, were synchronised in G1 with alpha factor, then released into media containing nocodazole. Sister-chromatid separation was scored as the percentage of cells containing two clearly separated GFP spots.

We introduced extra copies of *MAD3* and the *mad3-ken* mutants, expressed from the *MAD3* promoter and integrated at the *TRP1* locus, into a strain containing a wild-type *MAD3* gene. Quantitative western blotting revealed that the strains shown expressed ∼4-fold the wild-type level of Mad3p (Fig. 6A), and we found that all of these strains were sensitive to benomyl (Fig. 6B). Many transformants were analysed in this manner, and were found to have different expression levels of Mad3p (reflecting different copy numbers of integrated constructs). It was clear that increasing the level of Mad3p expression leads to increased benomyl sensitivity (data not shown).

**Figure 7:**
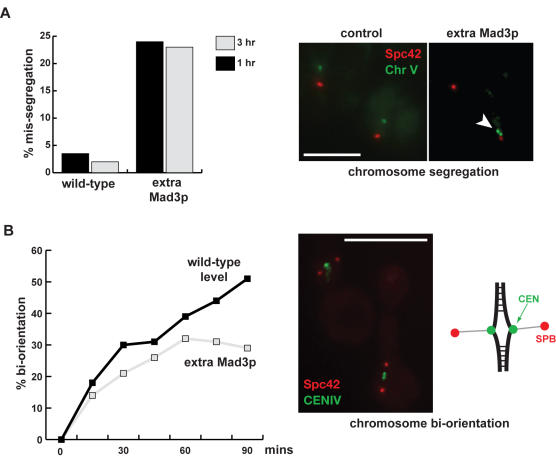
Over-expression of Mad3p induces bi-orientation and chromosome segregation defects. A) Mad3p overexpression induces chromosome mis-segregation following checkpoint “challenge”. Strains were pre-synchronised with α-factor, released and then arrested with nocodazole. The nocodazole was then washed out and cells arrested in the next G1. Cells were fixed and analysed for the presence of GFP-marked chromosomes. Chromosome V was labelled with GFP and spindle poles with SPC42-mCherry. Arrowheads mark G1 cells containing two copies of chromosome V. B) Mad3p overexpression induces chromosome bi-orientation defects. Cells were pre-synchronised with α-factor, then depleted for Cdc20p through the addition of Methionine. Cells were then released from G1 into media containing nocodazole and benomyl. The microtubule drugs were then washed out to allow soindle assembly, but Cdc20p remained represed to arrest cells in metaphase. Cells were briefly fixed and scored for bi-orientation (breathing centromeres). Centromere IV was labelled with GFP and spindle poles with *SPC42*-tomato. Scale bars are 5 microns.

Such sensitivity to anti-microtubule drugs could be for a number of reasons, including a defective spindle checkpoint response. We tested whether the strains with excess Mad3p could checkpoint arrest. Cells were pre-synchronised in G1 with alpha-factor, then washed and released into nocodazole-containing media. Analysis of GFP-marked chromosomes indicated that all strains were able to maintain sister-chromatid cohesion relatively efficiently (Fig. 6D). Yet, when we scored the same cultures for viability it was clear that, even though they were able to arrest, they died after 2–4 hours in nocodazole (Fig. 6C). We reasoned that the drug sensitivity might be due to an inability to recover properly from the checkpoint arrest. Therefore we quantitated the ability of strains overexpressing *MAD3* to accurately segregate chromosomes in the anaphase following the checkpoint arrest. To do this we pre-synchronised cultures in G1 with alpha factor, released into nocodazole media for 3 hours, and then washed out the nocodazole. We then imaged both spindle poles (SPC42-mCherry) and GFP-marked chromosomes during the ensuing anaphase (Fig. 7A). This experiment demonstrated that the Mad3p-overexpressing strains exhibit chromosome mis-segregation (25–40% for chromosome V), following release from the checkpoint arrest. Such a level of chromosome mis-segregation is significant enough to explain their benomyl-sensitivity.

To test whether this chromosome mis-segregation was due to a defect in chromosome bi-orientation we employed a strain containing a GFP-marked centromere. This strain has *CDC20* under control of the *MET* promoter, expresses the GFP-Tet repressor and has CENIV “GFP-marked” with an array of Tet operators, and has its SPBs marked with Spc42-tomato. If a replicated chromosome is bi-oriented at metaphase two GFP spots will be observed because the sister centromeres will be pulled apart (centromere breathing) by opposing forces from the two spindle poles [36]. Mono-oriented or unattached chromosomes are not pulled to both poles simultaneously and display a single spot (see Fig. 7B). Cells were synchronised with alpha factor in G1, and then Cdc20p was depleted by the addition of methionine to the media. Cells were then released from G1 into media containing nocodazole for two hours, enabling them to progress to metaphase. The nocodazole was then washed out, allowing cells to re-form a spindle, but *CDC20* was still repressed to maintain the metaphase arrest. Cells were fixed at 15 minute time points after nocodazole removal and scored for bi-orientation. An excess of Mad3p led to significant bi-orientation defects during recovery from checkpoint arrest: only 25–30% of cells displayed paired spots after 90 minutes, compared to 50–60% for control strains (Fig. 7B). This effect was not significantly affected by mutation of either KEN box (data not shown). Thus, overexpression of Mad3p perturbs chromosome bi-orientation and leads to significant chromosome loss during recovery from checkpoint arrest.

## Discussion

Here we have identified the N-terminal Mad3p KEN box as a critical link in MCC formation. Interestingly its mutation not only abolishes the MCC and spindle checkpoint function, but also leads to a striking mitotic stabilisation of the Cdc20p checkpoint effector. The same KEN box then appears to act as a Mad3p degron during late mitosis/G1, and we have shown that this Mad3p turnover is dependent on Cdh1-APC/C activity. Overexpression of Mad3p leads to anti-microtubule drug sensitivity, but this is not due to an inactive checkpoint. Rather, excess Mad3p induces bi-orientation defects, which lead to high levels of chromosome mis-segregation during recovery from spindle checkpoint arrest. Thus, the levels of both Cdc20p and Mad3p are subject to careful regulation, and this is important to ensure efficient checkpoint function and high fidelity chromosome segregation.

### Mad3p is degraded in a Cdh1-APC/C dependent manner

It has recently been shown that the Mps1 protein kinase is an APC/C substrate. Degradation of Mps1p in anaphase is necessary to prevent the spindle checkpoint from re-activation once sister chromatids separate and are no longer under tension [37]. Mps1p turnover was shown to be dependent on its three D boxes, on Cdc20p in anaphase, and on Cdh1p in G1.

Here we have demonstrated that Mad3p is another component of the spindle checkpoint that is degraded in an APC/C dependent manner. We do not believe that Mad3p degradation is critical to turn off the spindle checkpoint, although it is possible that it could play a role in checkpoint adaptation, and this is currently being explored. Our data is consistent with Mad3p being a Cdh1-APC/C substrate and the N-terminal KEN box acting as a degron. Further experiments are needed to prove this, such as *in vitro* ubiquitination assays demonstrating that Mad3p is poly-ubiquitinated by the APC/C in a KEN30-dependent fashion. We believe that the major role of its N-terminal KEN box is to enable Mad3p to bind to Cdc20p. This Mad3p-Cdc20p interaction, which is also dependent on Mad2p [28], is critical for the action of both Mad3p and Mad2p as *in vivo* anaphase inhibitors.

### The Mad3p KEN boxes are both required for spindle checkpoint function

**Figure 8:**
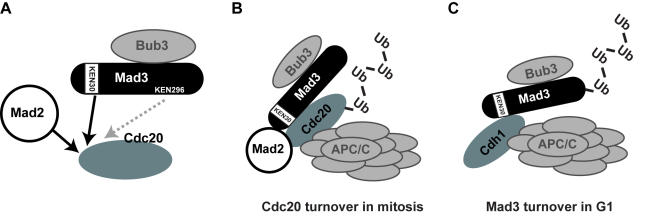
Models of Mad3p KEN box interactions. a) Mad3 KEN box interactions. Mad2p and Mad3-KEN30 are both required for stable Mad3p-Cdc20p binding. b) Cdc20p turnover in mitosis: this is dependent on Mad3-KEN30, and Mad2p, suggesting that this Mad3 KEN box acts “in trans” as a Cdc20p degron. c) Mad3p turnover in G1. Mad3p is degraded in a Mad3-KEN30, Cdh1, and APC/C dependent manner.

We have demonstrated that both of the conserved KEN boxes are important for Mad3p checkpoint function. The N-terminal KEN box is required for MCC formation and for Cdc20p turnover in early mitosis, as well as for Mad3p turnover in G1 (see Fig. 8). It has previously been shown that the two destruction boxes found in Cdc20p are not required for its turnover in early mitosis [14]. Our data suggests a model in which the Mad3 N-terminal KEN box acts *in trans* to regulate Cdc20p turnover in mitosis (see Fig. 8B). This could simply be because this KEN box is required for MCC formation, or because it acts *in trans* as a degron. Further work is necessary to distinguish these possibilities. Lack of other checkpoint proteins also affects Cdc20p turnover (data not shown and [14]), but lack of Mad3p has the most stabilising effect. These mutations are all known to affect the level of the MCC [28]. Such data suggests that it is a Mad3p-Cdc20p complex, most likely the MCC, which is recognised by the APC/C. Presumably Cdc20p is poly-ubiquitinated more efficiently than Mad3p, and this leads to its relatively short mitotic half-life (∼7 minutes for Cdc20p, rather than ∼90 minutes for Mad3p, see Fig. 5B).

There is significantly more Mad2p-Cdc20p in budding yeast extracts than there is MCC [38]. However, the role of the Mad2p-Cdc20p complex remains unclear, as it appears to be insufficient to act as an anaphase inhibitor *in vivo*. In many systems including fission yeast, Mad2p overexpression is sufficient for Cdc20p inhibition and metaphase arrest [39]. However, whilst this arrest is independent of most checkpoint components it does require Mad3p [31]. Mad2p is necessary for Mad3p to bind stably to Cdc20p [28], and we have now shown that the N-terminal KEN box of Mad3p is also critical for Cdc20p binding (see Fig. 8A). At present we are unable to functionally separate MCC formation from Cdc20p turnover, as both are abolished in *mad3-ken30* and neither are affected in *mad3-ken296*. Until such separation is achieved we are unable to assess the relative importance of these two pathways for Cdc20p inhibition. Interestingly, in the fission yeast *S.pombe,* whilst Slp1 is turned over in mitosis we find no evidence of Mad3p-dependence (*our unpublished data*). To our knowledge this has not been analysed in vertebrate systems, and it is possible that the checkpoint-dependent regulation of Cdc20p levels is specific to budding yeast mitosis.

However, the general importance of the N-terminal KEN box for checkpoint functions of Mad3p is well conserved. We have analysed equivalent *ken* mutations in *S. pombe* Mad3p with very similar results. Once again, the N-terminal KEN box is required for MCC formation, and the C-terminal motif is not (KGH, *manuscript submitted*). One striking difference from the budding yeast work presented here, is that in *S. pombe* we detect a stable interaction between Mad3p, Mad2p and mitotic APC/C. We found that this APC/C interaction is dependent on the N-terminal Mad3p KEN box and on Slp1p. We have found no evidence for stable Madp-APC/C binding in our budding yeast studies (data not shown, and see [40]).

In vertebrates a large BubR1-APC/C complex has recently been described in checkpoint-arrested cells [41]. Formation of that complex was shown to be dependent on an active checkpoint signal, and to be promoted by Bub1 and Aurora B kinase activities [41]. It will be interesting to see whether the BubR1-APC/C interaction is dependent on the N-terminal KEN box of BubR1. *In vitro* experiments with vertebrate proteins have demonstrated that BubR1 has multiple Cdc20 binding interactions, that this binding is dependent on Mad2, and that mutation of the N-terminus of BubR1 can abrogate checkpoint arrest [42]. These data are entirely consistent with our findings, and we conclude that the importance of the N-terminus of Mad3/BubR1, for MCC formation and checkpoint function, is conserved from yeast to human.

### Mad3p overproduction

The vertebrate Emi1 protein has recently been proposed to act as a pseudo-substrate inhibitor of the APC/C [43]. We tested whether *in vivo* Mad3p overproduction is sufficient to compete with Securin for Cdc20-APC/C. If so, Mad3p overexpression should induce a metaphase arrest. This was not the case, which argues against Mad3p acting as a simple pseudo-substrate inhibitor.

Why is Mad3p subject to Cdh1-APC/C dependent turnover? We found that a four-fold over-expression of wild-type Mad3p (and of the *ken* mutants) was sufficient to perturb mitosis following anti-microtubule drug treatment. Note, we carefully chose transformants expressing the same level of Mad3p, so any stabilising effect of the *ken30* mutation would not be apparent in our overexpression analysis. We provide evidence that the chromosome mis-segregation observed was due to defects in chromosome bi-orientation following spindle checkpoint arrest (Fig. 7). RNAi studies have suggested that loss of human BubR1 perturbs the regulation of kinetochore-microtubule attachments, possibly by interfering with Aurora B kinase activity [44]. It is tempting to speculate that Mad3p over-expression could also perturb Ipl1p (aurora) activities at budding yeast kinetochores. Alternatively, excess Mad3p could perturb Bub1p functions at kinetochores, most simply through competition for Bub3p. Experiments are underway to test such possibilities, but it is clear that Mad3p levels need to be carefully regulated to ensure efficient bi-orientation and checkpoint signalling. We believe that these Mad3p over-expression findings are relevant to human disease. Loss of BubR1 checkpoint function is lethal in the mouse [45], and biallelic BubR1 mutations are associated with childhood cancer and aneuploidy in humans [46]. In addition, it has recently been shown that Mad2 overexpression promotes aneuploidy and tumorigenesis in mice [47]. Our Mad3p studies suggest that overexpression, or dominant mutation, of human BubR1 could perturb checkpoint function or chromosome biorientation and thereby lead to the generation of aneuploidy.

In summary, we have shown that the conserved Mad3p KEN boxes are major determinants of checkpoint-effector (Cdc20p) interaction. We propose that these molecular interactions are central to the mode(s) of Cdc20p-APC/C inhibition by the spindle checkpoint. It is now time to integrate these Mad3/BubR1 findings with the checkpoint models that have been generated from structural studies of Mad2, Mad1 and Cdc20 [22], [25]. Recent advances in the structural analysis of the Bub proteins are a significant step in this direction [48]–[51]. In addition, we have shown that the N-terminal Mad3 KEN box is an important determinant of the stability of both Mad3p and Cdc20p. The levels of both of these proteins are very carefully regulated in yeast. If there is too much Cdc20p the spindle checkpoint is unable to efficiently inhibit it [8], [14], and if there is too much Mad3p cells display bi-orientation defects. Either of these would have significant consequences for the cell, and could be relevant to the generation of aneuploidy in human disease due to BubRI defects [46].

## Materials and Methods

### Yeast strains, media and standard techniques

All yeast strains used derivatives of W303 (*ade2-1 his3-11,15 leu2-3,112 trp1-1 ura3-1 can1-100 ssd1-d2*) and are listed in Table 1.

**Table 1:** 

KH34	a	*ade2-1 his3-11,15 leu2-3,112 trp1-1 ura3-1*
KH175	a	*mad3Δ.2 ade2-1 his3-11,15 leu2-3,112 trp1-1 ura3-1*
SJ135	a	*URA3::GAL1-MAD3 mad3Δ.2 ade2-1 his3-11,15 leu2-3,112 trp1-1*
SJ155	a	*URA3::GAL1-MAD3 cdc16-123 MCD1-6HA ade2-1 his3-11,15 leu2-3,112 trp1-1*
SJ147	a	*URA3::GAL1-MAD3 cdh1Δ::HIS3 ade2-1 his3-11,15 leu2-3,112 trp1-1*
SJ144	a	*URA3::GAL1-MAD3 cdc4-1 ade2-1 his3-11,15 leu2-3,112 trp1-1*
SJ146	a	*URA3::GAL1-MAD3 cdc34-2 ade2-1 his3-11,15 leu2-3,112 trp1-1*
SJ134	a	*URA3::GAL1-mad3-KEN30AAA mad3Δ.2 ade2-1 his3-11,15 leu2-3,112*
SJ136	a	*URA3::GAL1-mad3-KEN296AAA mad3Δ.2 ade2-1 his3-11,15 leu2-3,112*
KH375	a	*mad3Δ::URA3 TRP1::MAD3 ade2-1 his3-11,15 leu2-3,112 ura3-1*
KH376	a	*mad3Δ::URA3 TRP1::mad3KEN30AAA ade2-1 his3-11,15 leu2-3,112 ura3-1*
KH377	a	*mad3Δ::URA3 TRP1::mad3KEN296AAA ade2-1 his3-11,15 leu2-3,112 ura3-1*
KH378	a	*mad3Δ::URA3 TRP1::mad3KEN30/296AAA ade2-1 his3-11,15 leu2-3,112 ura3-1*
EK13	a	*mad3Δ.2 his3::CUP1prom-GFP-lacI12::HIS3 lacO(x256)::TRP1 ade2-1 leu2-3,112 ura3-1*
EK24	a	*mad3Δ.2 Pds1-Myc_13_ ade2-1 his3-11,15 leu2-3,112 trp1-1 ura3-1*
RJ10	a	*mad3Δ.2 Bub3-Myc_13_ ade2-1 his3-11,15 leu2-3,112 trp1-1 ura3-1*
LB04	a	*mad3Δ.2 Cdc20-Myc_13_ ade2-1 his3-11,15 leu2-3,112 trp1-1 ura3-1*
SJ161	a	*ura3::3xURA3tetO(x112) leu2::tetR-GFP::LEU2* *SPC42-mCherry::NAT ade2-1 his3-11,15 trp1-1*
KHC5	a	*TRP1::MAD3* *ura3::3xURA3tetO(x112) leu2::tetR-GFP::LEU2* *SPC42-mCherry::NAT ade2-1 his3-11,15 leu2-3,112*
SJ164	a	*TRP1::mad3-KEN30AAA* *ura3::3xURA3tetO(x112) leu2::tetR-GFP::LEU2* *SPC42-mCherry::NAT ade2-1 his3-11,15*
SJ165	a	*TRP1::mad3-KEN296AAA* *ura3::3xURA tetO(x112) leu2::tetR-GFP::LEU2* *SPC42-mCherry::NAT ade2-1 his3-11,15*
SJ166	a	*TRP1::mad3-KEN30/296AAA* *ura3::3xURA tetO(x112) leu2::tetR-GFP::LEU2* *SPC42-mCherry::NAT ade2-1 his3-11,15*
JF152	a	*URA3prom-tetR::GFP::LEU2 cenIV::tetO(x448)::URA3 METprom-CDC20 SPC42-Tomato::NAT ade2-1 his3-11,15 leu2-3,112 trp1-1 ura3-1*
SJ157	a	*TRP1::MAD3* *leu2*::*URA3prom-tetR::GFP::LEU2 cenIV:: tetO(x448)::URA3 ura3::URA3 METprom-CDC20 SPC42-Tomato::NAT ade2-1 his3-11,15*
SJ158	a	*TRP1::mad3-KEN30AAA* *leu2::URA3prom-tetR::GFP::LEU2 cenIV:: tetO(x448)::URA3 ura3::METprom-CDC20::URA3 SPC42-Tomato::NAT ade2-1 his3-11,15 leu2-3,112 ura3-1*
SJ159	a	*TRP1::mad3-*KEN296AAA *leu2::URA3prom-tetR::GFP::LEU2 cenIV:: tetO(x448)::URA3 ura3::METprom-CDC20::URA3 SPC42-Tomato::NAT ade2-1 his3-11,15 ura3-1*
SJ160	a	*TRP1::mad3-KEN30/296AAA leu2::URA3prom-tetR::GFP::LEU2 cenIV:: tetO(x448)::URA3 ura3::METprom-CDC20::URA3* *SPC42-Tomato::NAT ade2-1 his3-11,15 leu2-3,112 ura3-1*

Basic yeast methods and growth media and routine recombinant DNA methodology were performed as previously described [52], [53].

Fluorescent protein tagging was carried out using mCherry and tomato fluorescent proteins [54], [55].

### Mutation of KEN boxes

pKH535 is derived from pKH534 [28], which is YCplac22-based and contains the *MAD3* promoter. The *MAD3* ORF was amplified generating a *Bam*HI-*Eco*RI fragment and cloned in to pKH534. To make plasmids encoding the *mad3-ken* mutants we introduced point mutations into pKH535 using the Stratagene QuikChange method. These CEN-based plasmids were used to obtain the data in Fig. 3B,C and D.

To integrate *mad3* mutants into the genome, we excised a *Pml*I-*Eco*RI fragment (containing *CEN4, ARS1* and *TRP1*) from these mutant plasmids and replaced it with a *Pml*I-*Eco*RI fragment from YIplac204 (containing *TRP1*) to generate derivatives that could be targeted to the *TRP1* locus following linearization with *Bsg*I. These strains (KH375-378) were used in Fig. 3A.

### Generation of anti-Mad3p antibody

To obtain purified recombinant Mad3p we used the IMPACT-CN expression and purification system (New England Biolabs). *MAD3* was amplified from pKH535 and cloned into pKYB1 such that it was expressed as an intein-fusion protein. Protein expression and purification was performed according to the manufacturer's instructions, using a 36-hour self-cleavage reaction. Antiserum was affinity-purified as previously described [56]. Immunoblotting and immunoprecipitations were performed as previously described [56].

### Checkpoint assays

Benomyl sensitivity, rate of death and sister-chromatid separation assays were as previously described [57].

### Mitotic arrests

a number of approaches were used to arrest checkpoint-defective cells early in mitosis. As budding yeast assembles its spindle at the same time as replicating its DNA, we were able to use 10mg/ml hydroxyurea treatment to enrich for mitotic cells. For some experiments a combination of 10mg/ml hydroxyurea and 10 µg/ml nocodazole was used. Alternatively, checkpoint-deficient cells were arrested in metaphase by overexpression of non-degradable securin (*GAL-Pds1-Δdb*)–data not shown.

### Cycloheximide chase assays

Cells were synchronised in G1 through α-factor treatment in YEPRaff media, and *GAL-MAD3* was induced through the addition of 2% galactose to the media for 30 minutes. Cells were then washed in YPDA media to turn off the GAL promoter, and cycloheximide added to a final concentration of 1 mg/ml to prevent new protein synthesis. Time points were taken at 15 minute intervals and whole cell extracts immunoblotted with α-Mad3p antibodies. Film was scanned and bands quantitated using ImageQuant software, with dilution series of the zero time point being run as a control.

### Chromosome segregation

Cells containing Spc42-mCherry labelled SPBs and GFP-marked chromosome V were presynchronised in G1, and then released into media containing nocodazole (15 µg/ml) for 1 or 3 hours. The nocodazole was then washed out, and cells were arrested in the next G1 with a-factor. Cells were fixed for 5 minutes with 3.7% formaldehyde, washed and then imaged. Chromosome mis-segregation was scored as the % of cells that arrested in the next G1 with 2 GFP spots.

### Chromosome bi-orientation assay

Cells containing Spc42-tomato labelled SPBs, GFP-labelled CENIV, and Methionine-repressible *CDC20* were pre-synchronised in -Met media with α-factor. They were then washed and re-suspended in YPD supplemented with 8mM methionine and α-factor, and incubated for 2 hours to deplete cells of Cdc20p. The a-factor was then washed out and cells released into YPD (+8mM Methionine) media containing 30 µg/ml nocodazole and 30 µg/ml benomyl. 90 minutes later the nocodazole was washed out. At 15 minute intervals during the ensuing spindle assembly, at 30°C, cells were fixed and scored for bi-polar spindles with 2 paired GFP-spots due to “breathing” of the centromeric DNA [36]. Throughout each time-course 8mM Methionine was added every 30 minutes to ensure continued Cdc20p depletion. Bi-orientation was scored as the % of cells with breathing centromeres on short bi-polar spindles.

### Microscopy

cells were fixed briefly (5 mins) in 3.7% formaldehyde. They were then imaged using an Intelligent Imaging Innovations (3i) Marianas system, which incorporates a Zeiss Axiovert microscope, CoolSnap CCD, and Slidebook software.
